# Risk Stratification and Management of Intermediate-Risk Acute Pulmonary Embolism

**DOI:** 10.3390/jcm13010257

**Published:** 2024-01-02

**Authors:** Nichole Brunton, Robert McBane, Ana I. Casanegra, Damon E. Houghton, Dinu V. Balanescu, Sumera Ahmad, Sean Caples, Arashk Motiei, Stanislav Henkin

**Affiliations:** 1 Gonda Vascular Center, Mayo Clinic, Rochester, MN 55901, USA; brunton.nichole@mayo.edu (N.B.);; 2 Department of Cardiovascular Medicine, Mayo Clinic, Rochester, MN 55901, USA; 3 Division of Pulmonary and Critical Care Medicine, Mayo Clinic, Rochester, MN 55901, USA

**Keywords:** intermediate-risk pulmonary embolism, risk stratification, right ventricular dysfunction, thrombolytic therapy

## Abstract

Pulmonary embolism (PE) is the third most common cause of cardiovascular death and necessitates prompt, accurate risk assessment at initial diagnosis to guide treatment and reduce associated mortality. Intermediate-risk PE, defined as the presence of right ventricular (RV) dysfunction in the absence of hemodynamic compromise, carries a significant risk for adverse clinical outcomes and represents a unique diagnostic challenge. While small clinical trials have evaluated advanced treatment strategies beyond standard anticoagulation, such as thrombolytic or endovascular therapy, there remains continued debate on the optimal care for this patient population. Here, we review the most recent risk stratification models, highlighting differences between prediction scores and their limitations, and discuss the utility of serologic biomarkers and imaging modalities to detect right ventricular dysfunction. Additionally, we examine current treatment recommendations including anticoagulation strategies, use of thrombolytics at full and reduced doses, and utilization of invasive treatment options. Current knowledge gaps and ongoing studies are highlighted.

## 1. Introduction

Acute venous thromboembolism (VTE), presenting as pulmonary embolism (PE) or deep venous thrombosis (DVT), is a frequent cause of cardiovascular death worldwide, ranking third after myocardial infarction and stroke [1]. In longitudinal studies from the early 2000s to 2010s, the incidence of PE increased over time, with a concurrent increase in PE-related hospitalizations [2,3,4]. During this same timeframe, the case-fatality rates decreased in the United States and other developed countries. PE-related mortality is estimated from 19.4 to 32.3 per 100,000 individuals, with an in-hospital mortality rate of approximately 7% [5,6]. In patients with hemodynamic compromise due to PE, the reported mortality reaches 33%, often occurring suddenly or before therapy can be initiated [5].

Risk factors for the development of VTE include hospitalization, major surgery, fracture of the lower limb, trauma, spinal cord injury, cancer, hormonal therapy, pregnancy, autoimmune disease, presence of invasive lines, severe coronavirus disease (COVID-19), obesity, and thrombophilia [4,7,8]. It is well established that the incidence of VTE also increases with advancing age, with an estimated eight-fold increase for patients in the eighth decade of life compared to those in the fifth decade [4,6].

Due to the high morbidity and mortality associated with PE, early diagnosis and accurate risk assessment for hemodynamic compromise is vital to guide appropriate patient care. However, there is a vast spectrum of clinical presentations in acute PE, ranging from asymptomatic to obstructive shock with circulatory collapse. For this reason, acute PE has been further subdivided into classifications ranging from low-risk to high-risk, though these categories vary by societal guideline [4,9]. A growing body of literature has emerged in recent years investigating metrics, including serologic biomarkers and imaging parameters, to predict impending hemodynamic compromise. Studies have also focused on potential adjunctive therapies, such as mechanical thrombectomy and systemic or catheter-directed thrombolysis, to reduce mortality in this population.

Right ventricular (RV) failure is the main driver of mortality in PE, occurring as a consequence of acute RV pressure overload. In patients with high-risk PE, intervention with thrombolytics is essential, while those with low-risk PE can safely be managed conservatively with anticoagulation alone. However, an intermediate-risk (previously known as “submassive”) group demonstrates RV dysfunction without hemodynamic collapse, indicating increased risk for PE-related mortality. Accurately identifying this cohort is difficult due to the heterogeneous nature of patient symptoms, which may range from asymptomatic to dyspnea or chest pain. Recent studies have focused on identifying objective clinical markers and imaging parameters to quickly and accurately risk-stratify patients. In this review, we focus on current definitions of intermediate-risk PE, highlighting the benefits and limitations of available risk prediction models and discussing the utility of serologic biomarkers and imaging metrics of RV dysfunction, along with invasive hemodynamic assessment in this population. We also discuss escalation of care (EOC) therapies and the most recent results from thrombolytic trials, reviewing the controversial role of mechanical thrombectomy and alternative invasive procedures, as well as current knowledge gaps.

## 2. Acute Pulmonary Embolism Definitions and Classification

Acute pulmonary embolism may be classified based on the presence or absence of a provoking factor, symptoms, or acute hemodynamic instability, as well as the embolized material (e.g., thrombus, air, fat, tumor), its anatomic location and extent, and the risk of mortality. This review will focus on acute pulmonary embolism due to VTE and its classification based on risk models.

In 2011, the American Heart Association (AHA) published a scientific statement on the management of PE which outlined the classification of PE in three groups: low-risk, submassive, and massive [9]. Low-risk PE was defined as normotensive patients without biomarker elevation and normal RV function, while massive PE encompassed patients with sustained hypotension (systolic blood pressure < 90 mm Hg for 15 min), pulselessness, or profound bradycardia as a direct consequence of PE. Another group, deemed submassive, was defined as normotensive patients at an increased risk for adverse mortality outcomes. This patient population, while hemodynamically stable, had evidence of RV injury by serologic biomarker (troponin or natriuretic peptide), or RV dysfunction by imaging (RV dilatation on CT or echocardiography with an RV: LV ratio of >0.9), or new electrocardiographic changes (new right-bundle branch block, anteroseptal ST elevation depression, or anteroseptal T-wave inversion). These criteria were established from a body of studies conducted between 1999 to 2009 [4,10,11,12,13,14,15]. Simultaneously, two main clinical predictive scores were developed: the Geneva and the Pulmonary Embolism Severity Index (PESI), which supported these metrics as predictive for adverse outcomes in PE [9,11].

The European Society of Cardiology (ESC) released their guidelines, which remain the most frequently utilized for defining PE classifications, in 2019. As the understanding of RV dysfunction evolved, these updated guidelines aimed to accurately define metrics indicative of acute RV failure and identify additional risk factors that could predispose patients to poor clinical outcomes. The most notable difference in these guidelines when compared to the AHA 2011 definitions is the further subclassification of intermediate-risk to either intermediate-low or intermediate-high-risk (Table 1). This stratification developed from the realization that patients with submassive PE were still representative of a large and diverse patient group with a persistently high mortality rate, reportedly as high as 12.3% to 14.4% despite modern interventions [16,17].

In 2019, the AHA released an updated scientific statement specifically focused on interventions for PE. In this document, the differences between the ESC/AHA risk classification model were highlighted as inherently different due to the creation of the risk models for different purposes. However, for simplicity, the AHA adopted the classification of patients previously classified as “submassive” as an intermediate-risk group and patients previously classified as “massive” as a high-risk group. The 2019 AHA intermediate-risk group included all patients in the ESC intermediate-risk group (both intermediate-low-risk and intermediate-high-risk) [18].

In the ESC risk model, the Pulmonary Embolism Severity Index (PESI), either the original or simplified model (Table 2), is employed to distill clinical information, including vital signs and medical comorbidities, into a risk score. The PESI score was developed in 2005 and utilized 11 patient characteristics independently associated with increased mortality. These were used to stratify patients into five severity classes (I–V) ranging from very low-risk to very high-risk [19]. For patients in class I, the risk of inpatient death and complications was found to be <1%, and it ranged from 10–24.5% in class V. This is useful for rapidly identifying patients at risk for all-cause mortality at 30 days after PE diagnosis and was validated using both an internal and external validation cohort in the initial study [19]. In 2010, Jiménez et al. completed a derivation study simplifying this score (sPESI) to help quickly identify patients at an increased risk for 30-day mortality [10]. This is helpful for quick assessment of patient disposition but is not useful for nuanced risk category placement or analysis. It is worth remaining mindful of clinical characteristics influencing PESI variables, such as sepsis for example, and that sPESI has limited specificity in predicting mortality in high-risk patients.

Patients scoring as PESI I–II or with an sPESI of ≤1 are considered low-risk, and selected cases could be managed in the outpatient setting. This has been validated by additional studies demonstrating the safety of this strategy [21]. In patients with PESI class III–V or with an sPESI score of ≥1, further evaluation is necessary. Imaging parameters and cardiac serologic biomarkers are utilized to further classify these patients to predict PE-related mortality at 30 days (Table 2). Patients with both evidence of RV dysfunction by imaging and serologic biomarkers were defined as intermediate-high-risk, while patients with only one or neither metric fulfilled are classified as intermediate-low-risk. This was an important update from prior AHA statements because it served to identify a PE population at risk for hemodynamic compromise while also attempting to limit confounders such as chronic RV dysfunction from other causes.

The latest release of societal guidelines on the management of PE is the 2021 guidelines on Antithrombotic Therapy for VTE Disease from the American College of Chest Physicians (ACCP). These guidelines more broadly categorize PE on the basis of ‘associated with hypotension’ or ‘not associated with hypotension’ to guide next treatment steps [22]. However, a limitation of this model is its lack of inclusion of RV hemodynamic parameters. As such, the guidelines potentially exclude a population of PE patients at risk for hemodynamic compromise and place a higher burden on the clinician to differentiate between hypotension due to PE or due to other causes (e.g., cardiogenic shock, sepsis, etc.).

## 3. Indicators of Risk in Pulmonary Embolism

### 3.1. Serologic Biomarkers

It is crucial to highlight that any single serologic marker is not sufficient to detect risk for hemodynamic collapse and must be considered within the context of clinical history, baseline comorbid conditions, physical examination, and imaging parameters. Therefore, serologic markers of myocardial injury and right ventricular dysfunction can be useful in identifying patients at high risk for circulatory collapse when interpreted in the appropriate clinical setting.

The most widely used marker for myocardial injury is plasma troponin (T or I) level. In hemodynamically stable patients with PE, an elevated troponin level on presentation is associated with higher risk of mortality when compared to those with negative troponin (T or I) values [23,24]. Similar findings are reported with the employment of high-sensitivity troponin T (hsTnT) assays [17,23,24]. In small prospective trials, patients with higher baseline hsTnT values on presentation were noted to experience higher rates of adverse 30-day outcomes compared to those with low hsTnT values (defined as <14 pg/mL). Furthermore, the increased sensitivity of this assay led to the re-classification of nearly 50% of patients who otherwise would have been classified as low-risk by traditional AHA guidelines [17]. A post-hoc analysis from the Prognostic Value of Computed Tomography (PROTECT) trial compared outcomes of PE patients with conventional troponin elevation to patients with hsTnT elevation. When evaluating troponin as a binary metric, only conventional troponin elevation was associated with increased odds for hemodynamic collapse or all-cause death within 30 days of PE. Moreover, there were no reported adverse outcomes in patients with normal conventional troponin and elevated hsTNT [25]. This supports the addition of the intermediate-low-risk ESC classification, where subtle signs of myocardial injury can be detected but may not necessarily translate to the need for more aggressive interventions.

Serologic evaluation for ventricular dysfunction, due to increased RV pressure and myocardial stretch, includes B-type natriuretic peptide (BNP) and N-terminal (NT)-BNP. Again, prior meta-analyses support the prognostic value of elevated BNP and/or NT-proBNP, with elevated values conferring a higher risk for adverse clinical outcomes, including death [4,26]. Conversely, patients with low troponin values, BNP, or NT-proBNP levels are useful for identifying low-risk PE events with a high negative predictive value [4].

The difficulty with all of the aforementioned biomarkers is the lack of specificity for PE. While prognostically helpful for identifying higher-risk PE patients, these biomarkers can also be elevated independently from the presence of concurrent medical conditions such as chronic pulmonary hypertension, left-sided heart failure, volume overload in the context of renal failure, etc. A classic example of this confounding is the patient with left ventricular failure with volume overload with high NT-pro BNP, but who is incidentally found to have a single, subsegmental pulmonary embolism. However, in patients with known baseline values of these markers, in the absence of new clinical scenarios that may be associated with abnormal serologic markers, it is reasonable to attribute elevations to PE.

To summarize, troponin and BNP or NT-pro BNP are generally reliable parameters of acute RV dysfunction due to PE; however, interpretation of these values is difficult in the medically complex patient and makes curating an algorithm for the management of PE patients challenging. This is particularly true when attempting to identify intermediate-high-risk patients for invasive therapy strategies. Further studies are needed to understand the appropriate utilization of biomarkers in this population and if threshold values or delta from baseline values could be useful to provide additional insight.

### 3.2. Imaging: Use of Echocardiography, Computed Tomography Pulmonary Angiography, and Invasive Hemodynamic Assessment

Under normal physiologic circumstances, the RV functions with a low pulmonary vascular resistance (PVR) and, therefore, low afterload. In acute PE, the PVR increases quickly and results in a rapid increase in RV systolic pressure, causing pressure and volume overload and RV dysfunction. This can lead to overt RV failure in severe cases [27]. Increased pulmonary artery pressure (PAP) occurs when at least 30% to 50% of the pulmonary arterial vasculature is occluded from thromboemboli [4]. RV dysfunction and volume overload can be appreciated on imaging by echocardiography or computed tomography pulmonary angiography (CTPA) in at least half of patients hospitalized for PE [28]. These imaging findings serve as important prognostic markers for patients with intermediate-risk PE but can be challenging to quantify due to the irregular shape of the RV, which limits the utility of a single metric to quantify severity of dysfunction. When carefully utilized, echocardiography (ECHO) parameters of RV dysfunction can provide helpful insight into cardiac function to help prognosticate mortality in patients with intermediate-risk PE [29]. It is important to note that signs of RV dysfunction on imaging are not specific to acute PE alone and may be present in patients without PE. Therefore, these must be evaluated in the context of the patient’s baseline cardiac function, other medical comorbidities, and interpreted with caution due to variations in techniques.

Standardization of RV function on echocardiography by the American Society of Echocardiography (ASE) did not occur until 2010. As a result, early echocardiography studies utilized an array of measurements to define RV dysfunction [30]. Despite this challenge, early meta-analyses did show a 2.29-fold increase (95% CI 1.61–3.26) in short-term mortality for hemodynamically stable patients with evidence of RV dysfunction on echocardiography [30]. Frequently utilized measures include RV cavity dilatation, diminished inspiratory phase of a distended inferior vena cava (IVC), and elevated RV to left ventricle (LV) ratio (>1.0). RV dilatation is considered a hallmark for PE and has been reported in >25% of PE cases [27,31,32].

Frequently on both CTPA and ECHO assessment, a comparison of RV to LV size is made on imaging, with a ratio of ≥ 0.9 accompanied by an underfilling LV being suggestive of PE. While the RV:LV ratio remains one of the most frequently cited metrics for RV dysfunction in the medical literature, there are significant variations in calculation that impact the diagnostic accuracy of this measurement. This occurs due to different methods of measurement, utilizing the epicardial border (outer edge-to-outer edge) or endocardial border (inner edge-to-inner edge), timing of the measurement (end-diastole versus end-systole), and use of a gated image. For this reason, the RV:LV ratio may not always be a reliable metric of RV dysfunction. One study demonstrated that when studied in isolation in patients with low-risk PE, an RV:LV ratio of >1.0 on imaging did not carry significant risk for mortality, recurrent PE, or total adverse events at 3 months [32].

**Figure 1:**
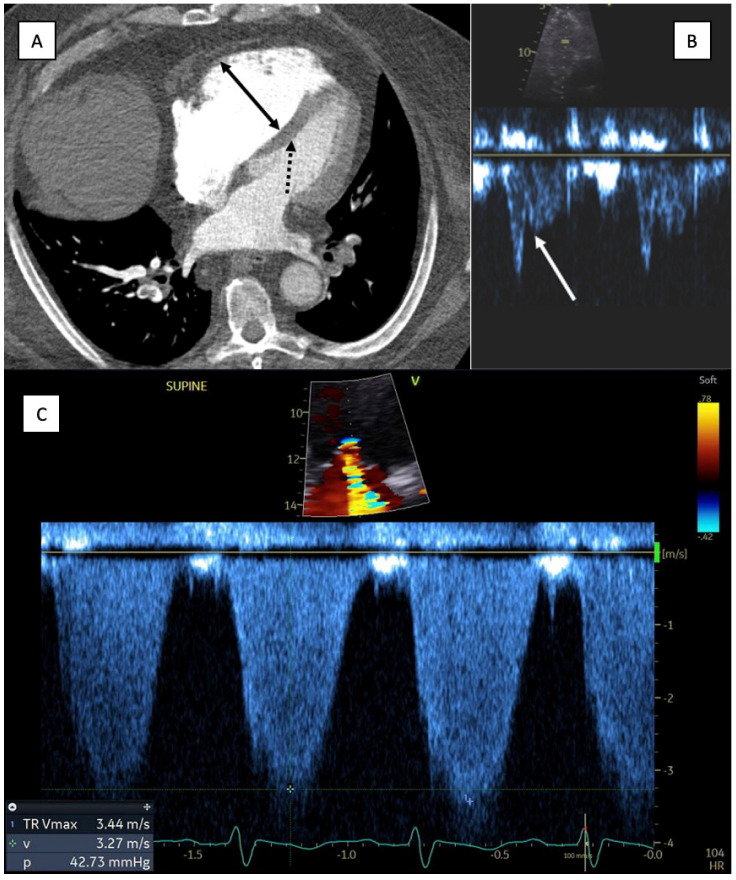
Computed tomography angiography findings consistent with right ventricular dysfunction [32]. (**A**) CTA chest: Axial image at the level of the mitral valve. Solid arrow demonstrating enlargement of the right ventricle compared to the left ventricular cavity. Dashed arrow highlights the deviation of the interventricular septum towards the left ventricle. (**B**) Transthoracic ECHO (same patient): shortened pulmonary ejection acceleration time (AcT) with a “notched” midsystolic velocity deceleration in the RV outflow track (white arrow). (**C**) Transthoracic ECHO (same patient): demonstrating tricuspid regurgitation (TR) peak systolic gradient (TRPG) of less than 60 mm Hg (42.7 mm Hg), consistent with the proposed 60/60 sign.

For CTPA, the simplest and accepted way to identify RV dysfunction is by assessing the axial plane where ventricular cavities are maximally visualized, typically at the plane of the mitral valve. RV dysfunction is considered dilated if the RV cavity is wider than the LV cavity. Another CTA finding consistent with RV dysfunction is the deviation of the interventricular septum to the LV (Figure 1) [33]. Again, it is important to note that this method carries limitations in patients with pre-existing lung conditions or pulmonary hypertension with baseline RV enlargement. Other findings suggestive of RV dysfunction on CTPA include reflux of contrast into the IVC, interventricular septal deviation, assessment of pulmonary artery size, and presence of RV dilatation [34,35].

Echocardiographic metrics of RV dysfunction include the McConnell sign, whereby the free RV midwall becomes hypokinetic with hyperkinetic apical segment; a decreased RV tricuspid annular plan systolic excursion (TAPSE) of <16 mm; decreased peak systolic velocity of the tricuspid annulus (<9.5 cm/s); and the presence of interventricular septal bowing, which occurs in severe RV dysfunction near the point of hemodynamic collapse [27,34]. However, due to the asymmetric shape of the RV, basal segments and TAPSE may remain normal in cases of dysfunctional RV. Additionally, in individuals with baseline pulmonary hypertension with elevated PVR, RV hypertrophy can develop, reducing the sensitivity and predictive value of these measures [4,27,28]. For this reason, recent studies have attempted to develop a combination of echocardiographic findings to achieve a high positive predictive value for PE that can be utilized even in those with pre-existing cardiopulmonary pathology [4,31]. One proposed method is the “60/60” sign, defined as the presence of both a shortened pulmonary ejection acceleration time (AcT) of <60 ms with a “notched” midsystolic velocity deceleration in the RV outflow tract (RVOT) and reduced (<60 mmHg) tricuspid regurgitation peak systolic gradient (TRPG) (Figure 1). A single center analysis of 511 consecutive patients with PE reviewed echocardiograms in acute PE (all subtypes) for presence of the McConnell sign, the “60/60” sign, and the presence of right heart thrombus, occurring in 19.8%, 12.9%, and 1.8% of patients, respectively. These rates increased dramatically in a subgroup analysis for high-risk PE patients, with a reported prevalence of 75% with the McConnell sign and 31.2% with the 60/60 sign. Conventional metrics of RV dysfunction (RV:LV ratio of ≥0.9) were identified in only 20% of all patients included in the study [31]. It is important to note that studies utilize varying definitions of RV dysfunction, commonly using either an RV:LV ratio of ≥0.9 or ≥1.0. Prior meta-analyses have demonstrated an association between higher cut-offs of RV:LV ventricle ratio with a higher risk of death [36]. It is unclear which ratio should be employed routinely, though our group utilizes RV:LV > 0.9 to increase sensitivity and in accordance with previously published guidelines [9].

Additional studies have demonstrated the RVOT velocity time integral (VTI), an echocardiographic surrogate for stroke volume, as a significant predictor for invasively derived low cardiac index (CI) and risk of mortality related to PE [29,36]. Specifically, in a small study, an RVOT VTI of <9.5 cm was associated with higher PE-related mortality (13.6%) when compared to patients with an RVOT > 9.5 cm (1.28%), though all-cause mortality between the groups was not significantly differently [29]. Three-dimensional echocardiographic assessment of global and regional RV strain in patients with intermediate-risk PE may provide additive fidelity for patients at risk for hemodynamic compromise, though further studies are needed to explore this [37].

For patients with intermediate-risk PE undergoing endovascular intervention, hemodynamic assessment by right heart catheterization can further provide insights about mortality risk. A CI < 2.2 L/min/m^2^ has been associated with increased risk for PE-related mortality [29]. In small studies, approximately half of patients undergoing endovascular intervention are noted to have reduced CI on hemodynamic assessment despite being normotensive [29,38]. However, this metric was not utilized in the early interventional trials for risk stratification. Future studies may be helpful to fully ascertain if this cohort derives additive benefit from more aggressive management strategies.

When invasive hemodynamic assessment is pursued, a clearer understanding of the hemodynamic consequences of a PE can be obtained through evaluation of right ventricle to pulmonary artery (RV-PA) coupling [39]. Simply stated, the RV is able to accommodate for increased afterload from the pulmonary artery (PA) to a certain degree; however, there is a point in which adaptive mechanisms are no longer able to compensate. This leads to an ‘uncoupling’ between the RV-PA, which signals RV decompensation [40,41]. The gold standard for RV-PA assessment is by invasive catheterization and is calculated by the ratio between the RV end-systolic elastance (Ees) and the pulmonary arterial elastance (Ea). When the RV begins to decompensate, a decline is noted in the Ees, resulting in a decline in the Ees/Ea, implying uncoupling of the RV-PA [39,40]. There has been recent effort to derive this metric non-invasively by echocardiography. Examples include using surrogate markers of the tricuspid annular plane systolic excursion (TAPSE) to systolic pulmonary artery pressure (PASP) ratio, right ventricular ejection fraction (RVEF) to PASP, or right ventricular fractional area change (RVFAC) to right ventricular systolic pressure (RVSP) [39]. Further studies are required to fully understand the reproducibility and feasibility of these metrics, since good-quality images are essential for calculation. Further trials are also needed to determine clinically significant Ees/Ea values and thresholds for intervention.

## 4. Management of Intermediate-Risk Pulmonary Embolism

### Medical Management: Anticoagulation

The cornerstone of acute medical management for PE is anticoagulation [42]. Initial parenteral therapy is recommended with low-molecular weight heparin (LMWH) above unfractionated heparin (UH) infusion due to the rapid rise of therapeutic drug levels and decreased risk of heparin-induced thrombocytopenia. Furthermore, a meta-analysis demonstrated improved outcomes in PE patients initially treated with LMWH compared to UH, including a reduction in thrombotic complications, an increased safety profile for major hemorrhage, and lower risk of mortality [43]. Heparin infusions should be considered if there is concern for impending hemodynamic compromise and consideration for imminent endovascular intervention.

When stabilized and appropriate for discharge, direct oral anticoagulant (DOAC) therapy is frequently utilized due to ease of use. The safety and non-inferiority to vitamin K antagonists (VKA) of the direct oral anticoagulant (DOAC) agents is well established for PE treatment and incorporated in current practice guidelines [44,45,46]. In review of the major DOAC trials for thromboembolism, despite frequent DOAC utilization for treatment of intermediate-risk PE, the Hokusai-VTE investigators were the only group to specifically review the use of edoxaban in patients with right-ventricular dysfunction [47]. There was a reduction in VTE recurrence for patients with RV dysfunction treated with edoxaban compared to warfarin [47]. Important considerations for DOAC use include renal or hepatic function, as these agents are not well studied in patients with end-stage renal disease and should be avoided in patients with underlying hepatic dysfunction beyond Child–Pugh Class A. Patient history of bariatric surgery, potential medication interactions, patient weight, and financial feasibility should also be considered prior to prescription of DOAC therapy for first-line management of intermediate-risk PE, as these components can lead to subtherapeutic drug levels or non-compliance. Recent guidelines by the International Society on Thrombosis and Haemostasis support the use of apixaban and rivaroxaban for patients with a BMI > 35 or body weight > 120 kg; however, data are limited to support use in patients at weight extremes (BMI > 50 or body weight > 150 kg). The use of dabigatran or edoxaban in patients with a BMI > 35 or body weight > 120 kg has not been sufficiently studied and is not recommended [48]. For patients with unprovoked PE, antiphospholipid syndrome (APS) should be considered prior to dismissal on DOAC therapy, as patients with APS are known to have higher risk for DOAC failure. Additional serologic evaluation for inherited thrombophilias is not required prior to DOAC use in most patients. DOACs have also shown their effectiveness in cancer-associated PE and should be considered first-line in this population where traditionally LMWH has been used preferentially [49].

In patients with clinical contraindications or financial barriers to the use of DOAC therapies, anticoagulation with VKA is recommended. When used, monitoring for VKA therapy should be completed using international normalized ratio (INR) values with an anticipated range of 2.0–3.0. When VKA therapy is initiated, patients with acute VTE require bridging with LMWH until a therapeutic INR level can be achieved and for a minimum of 5 days. It is advised to treat with LMWH if the INR is below two during the first month after the event. A typical treatment course with therapeutic anticoagulation after intermediate-risk pulmonary embolism ranges from 3–6 months. Patients with unprovoked PE or patients with cancer-associated venous VTE should be strongly considered for extended VTE prophylaxis depending on the clinical scenario.

## 5. Escalation of Care Therapies

### 5.1. Role of Thrombolytic Therapy

In patients with concerning features for impending hemodynamic compromise, anticoagulation alone may not be a sufficient strategy to prevent clinical decline. The “Fibrinolysis for Patients with Intermediate-Risk Pulmonary Embolism” (PEITHO trial) was completed in 2014 to evaluate the use of tenecteplase (ranging from 30 to 50 mg) plus anticoagulation compared to placebo plus anticoagulation in a double-blind control study. Primary outcomes for this study included death or hemodynamic collapse within 7 days of enrollment, with a safety endpoint of major bleeding or stroke [50]. Although hemodynamic collapse in patients treated with tenecteplase plus heparin occurred less frequently when compared to the placebo group (2.6% vs. 5.6%, OR 0.44), this did not provide a net benefit at 30 days due to the increased risk of extracranial bleeding (6.3% vs. 1.2%) and hemorrhagic stroke in the tenecteplase group [50].

Due to concern for increased bleeding risk, the “Moderate Pulmonary Embolism Treated with Thrombolysis” (MOPETT trial) was conducted using low-dose tissue plasminogen activator (tPA; 0.5 mg/kg with maximum dose of 50 mg). The primary endpoints for this study were pulmonary hypertension, defined as a PASP of ≥40 mm Hg on echocardiogram, and a composite of pulmonary hypertension and recurrent PE after 28 months of follow-up. Pulmonary hypertension was noted in 16% of the low-dose thrombolytic group compared to 57% of the control group. Hospital duration was shorter for patients receiving low-dose thrombolytics (2.2 days vs. 4.9 days). A significant difference for death or PE recurrence was not appreciated in this study cohort. Therefore, low-dose thrombolytics are postulated to be safe; however, this has not yet been shown to alter other clinical outcomes [51]. It should be noted that this trial did include a small sample size. Conversely, a retrospective study comparing half-dose atleplase (50 mg) to full-dose (100 mg) suggested similar mortality rates and major bleeding events [52]. In summary, further large trials are needed to better ascertain the role of low-dose lytic therapy in this population.

Long-term outcome data for patients with intermediate-high-risk PE and thrombolytic therapy are limited. In 2017, the PEITHO group published long-term data in a subgroup with a median follow-up time of 37.8 months after systemic thrombolytic therapy. Survival was similar between the systemic thrombolysis and placebo groups (20.3% vs. 18.0%, respectively). Functional limitations and persistent dyspnea were similar between the two groups as well, approximately 33% in both arms. There was not a significant difference between echocardiographic findings for pulmonary hypertension or persistent RV dysfunction at follow-up [53]. To summarize, there was no significant difference for mortality, functional outcomes, or echocardiographic metrics to suggest long-term improvement for patients with intermediate-risk PE receiving thrombolytics.

In summary, while systemic thrombolytic therapy has shown reductions in pulmonary arterial pressures and reduced hospitalization length, no current evidence signals further mortality benefit in patients with intermediate-risk PE. However, these trials are not completely reflective of our current understanding of intermediate-high-risk PE nor are reflective of current recommended treatment strategies [54]. For example, the MOPETT trial enrolled patients with metrics consistent with symptomatic PE; however, evidence of RV dysfunction by imaging or cardiac biomarkers was not necessary for study enrollment [51]. Even in the PEITHO trial, RV dysfunction was defined by either echocardiographic or CT parameters, with a right-to-left ventricular end-diastolic diameter of >0.9 or right-to-left ventricular diameter ratio of >0.9 on CT. As mentioned earlier, these are not ideal metrics to define RV dysfunction. This highlights the need for further investigation into the role of systemic thrombolytics and the need to refine the definition of RV dysfunction for patients included in these trials. Furthermore, while prior studies of thrombolytic agents in intermediate-risk PE provided conflicting results, a recent meta-analysis completed in 2023 suggests that there may be further short-term benefits, though the evidence is overall weak [54]. With the rapid evolution of PE management, many prior reviews of thrombolytics included studies with antiquated agents or substandard doses. When only studies with current thrombolytic agents and standard dosing were included, a meta-analysis by Mathew et al. found that patients receiving systemic thrombolytics compared to anticoagulation alone had a decreased need for vasopressor support (RR 0.27, 95% CI 0.11–0.64) and rescue thrombolysis (RR 0.25, 95% CI 0.14–0.45). This occurred at the expense of increased intracranial hemorrhage and did not yield a significant in-hospital mortality difference between patients receiving thrombolytics versus those managed with anticoagulation alone [54].

### 5.2. Catheter-Based Strategies

The benefit of catheter-based therapies in high-risk PE is becoming well established; however, impacts on outcomes in intermediate-risk PE remain unclear [55]. Current invasive management strategies for intermediate-risk PE include mechanical thrombectomy and catheter-directed thrombolytic (CDT) systems, including ultrasound-facilitated systems (US CDT), which appeared on the market in the past decade. Mechanical thrombectomy devices available for clot retrieval include the Inari Medical (Irvine, CA, USA) FlowTriever, the Penumbra (Alameda, CA, USA) Indigo Aspiration Thrombectomy System, and the Angiodynamics (Latham, NY, USA) AngioVasc/AlphaVac. Potential benefits to mechanical thrombectomy include a measurable hemodynamic response while patients are in the interventional suite, reduced bleeding risk comparative to systemic lytic therapy in the short term, significantly shorter length of hospital stay, potential deferral of intensive care unit admission, and more rapid improvement in RV hemodynamic parameters compared to anticoagulation [56,57,58]. However, drawbacks include the risk for vascular or cardiac injury as well as a prolonged procedural time, depending on the nature of the thrombus being extracted. On retrospective review, compared to systemic thrombolytic therapy, patients undergoing catheter-directed thrombolytic therapy carry similar risk for major bleeding (RR 0.52: 95% CI 0.37–1.76) but lower risk for in-hospital mortality (RR 0.52, 95% CI 0.40–0.68) as well as intracranial hemorrhage (RR 0.66, 95% CI 0.47–0.94) [59]. However, these data are limited based on the retrospective nature of this study and by the lack of further classification based on PE risk.

Catheter-based thrombolytic strategies include EKOS (Boston Scientific, Marlborough, MA, USA), Bashir endovascular catheter (Thrombolex, New Britain, PA, USA), or the use of standard infusion catheters. Benefits to CDT include shorter procedural time; however, patients are generally admitted to the intensive care unit and response to treatment requires a minimum of several hours, along with the risk of patient discomfort due to necessary prolonged supine positioning. Patients with active bleeding, head trauma, or cerebral infarction in the preceding 3 months or known intracranial tumors/aneurysms have contraindications for these strategies. Relative contraindications include trauma or surgery within the preceding 10 days, uncontrolled hypertension (systolic BP > 180 mm Hg or diastolic > 110 mm Hg), or gastrointestinal bleeding within the preceding three months [60].

Notable studies for catheter-directed thrombolysis and mechanical thrombectomy in intermediate-risk PE are summarized in Table 3. Importantly, while randomized controlled trials examining the use of endovascular interventions exist, all the existing device trials include only a small subset of patients, which makes extrapolation difficult. Furthermore, most PE device studies for intermediate-risk PE examine only metrics of RV improvement, typically quantified by the RV/LV ratio, modified Miller score (a score for radiographic extent of thrombus), or PASP alone. The recently published REAL-PE trial, a retrospective study, signals that major bleeding occurs more frequently with mechanical thrombectomy when compared to ultrasound-directed CDT, including higher rates of intracranial hemorrhage. While these results provide insight into the bleeding risk and safety of endovascular strategies, the findings are somewhat counterintuitive. This is likely due to the retrospective trial design and confounding bias in patient selection for mechanical thrombectomy [61].

To date, there remains a paucity of evidence to assess whether these acute hemodynamic changes improve clinical outcomes for patients, particularly as they pertain to the development of chronic thromboembolic pulmonary hypertension (CTEPH) or quality of life. It is also unclear whether there is a subset in the intermediate-risk PE population who may benefit from catheter-based therapies over others. For example, when analyzing patients with intermediate-high-risk PE, retrospective data suggest a mortality and bleeding benefit. Furthermore, an analysis of the National Inpatient Sample of cancer patients with intermediate or high-risk PE also suggested improved mortality, although higher bleeding [70]. Further data are urgently needed to prospectively analyze catheter-based therapies, particularly in the intermediate-high-risk PE population and its subsets.

## 6. Ongoing Trials for Intermediate-Risk Pulmonary Embolism

Current ongoing clinical trials include the Higher-Risk Pulmonary Embolism Thrombolysis (HI-PEITHO) study, which is currently enrolling patients with intermediate-high-risk PE with increased risk of death or hemodynamic compromise. This study aims to compare composite clinical outcomes at 7 days for patients receiving ultrasound-facilitated catheter-directed thrombolytic therapy with anticoagulation versus anticoagulation alone. Additional aims of the study include comparison of patients’ functional status, quality of life indicators, and health-care utilization in the subsequent 30 days, 6 months, and 12 months after index PE [63].

The PE-TRACT study is another new multi-center randomized controlled trial investigating the use of catheter-directed thrombolytic (CDT) therapy in addition to standard anticoagulation compared to anticoagulation alone in patients with intermediate-risk PE. The anticipated enrollment will include 500 patients with an anticipated 6-year follow-up. The goal of this study is to examine routine use of CDT in patients with intermediate-risk PE and could also provide new insight into the natural history of patients with intermediate PE [71].

Ongoing device trials include the FLASH and PEERLESS trials, examining the use of Inari FlowTriever systems in both intermediate and high-risk PE, respectively. The FlowTriever All-Comer Registry for Patient Safety and Hemodynamics (FLASH) study aims to compare safety outcomes at 48 h for patients undergoing thrombectomy with the Inari Flowtriever system compared to patients receiving conservative therapy with anticoagulation alone. Interim analysis of the initial 250 patients enrolled demonstrated a small number of major adverse events (1.2%) in the Inari group, which all resolved without sequelae. Intraprocedural hemodynamic improvements were also reported, with an average reduction of mean pulmonary artery pressure of 7.1 mmHg with patient-reported symptomatic improvement [66]. The PEERLESS trial, also utilizing the Inari FlowTriever system, is an ongoing randomized controlled study comparing intermediate-high-risk PE patients treated with mechanical thrombectomy (FlowTriever System) versus catheter-directed thrombolysis [67]. In May 2023, the Inari Medical group additionally announced its intention to start the PEERLESS II trial. As an expansion of the PEERLESS trial, PEERLESS II is another randomized controlled trial aiming to compare outcomes of patients with intermediate-risk PE treated with the FlowTriever system compared to those treated with anticoagulation alone [72]. A summary of prior and ongoing device trials is summarized in Table 3.

## 7. The Importance of Multidisciplinary Teams: The Pert

**Figure 2:**
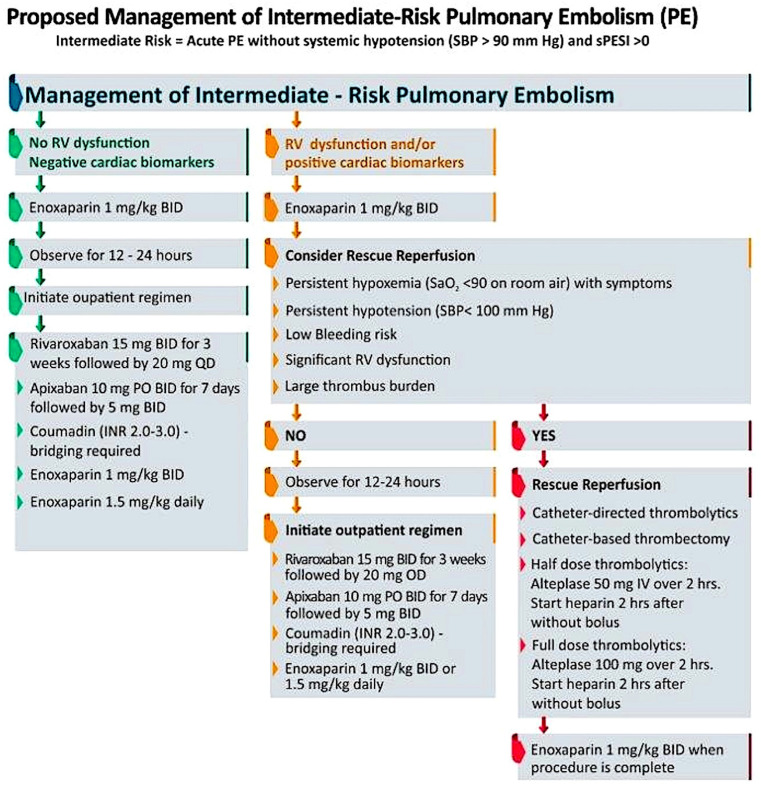
Decision–making in management of intermediate–risk pulmonary embolism.

Given the high complexity, mortality risk, and evolving nature of available therapies for patients with intermediate-risk PE, a multidisciplinary team approach is crucial. Since 2012, Pulmonary Embolism Response Teams (PERTs) have become common at many institutions to streamline rapid assessment along with prompt implementation of EOC therapies for patients with intermediate or high-risk PE. Given the heterogeneity of hospitals, PERTs vary in composition between institutions, but generally consist of multidisciplinary teams including pulmonary critical care, cardiology, vascular medicine, interventional radiology, and interventional cardiology [1]. In the sentinel paper from Massachusetts General Hospital, the implementation of the PERT was rapidly adopted nationwide. Systemic anticoagulation was the primary treatment modality at the time of publication in 2016 [1,73]. However, since publication, catheter-directed therapies have rapidly developed and become accessible, strengthening the necessity for PERTs to assist in the nuanced decision-making for this population. Recent reviews of PERTs have found a decrease in ICU length of stay, reduced bleeding rates, decreased utilization of IVC filter placement, and short time-to-therapeutic anticoagulation when compared to historical controls [1,74,75,76]. It has been hypothesized that PERTs may reduce PE-related mortality; however, results are conflicting [1,74,76]. This may be in part due to the observational nature of some studies (pre- and post-PERT) which do not account for changing guidelines and therapies occurring simultaneously [1,74]. It is important to note that the expansion of PERTs across the nation has flourished. Although this expansion has facilitated interventional procedures, it is also important to recognize that the primary role of the PERT team should also be to carefully assess each patient and de-escalate management where bleeding risk predominates. There is additional benefit in a careful comprehensive assessment to exclude pre-existing pathologies which can confound the clinical presentation. A recommended outline for approaching a patient with intermediate-risk PE is summarized in Figure 2.

## 8. Conclusions

The current understanding of intermediate-risk, previously submassive, PE is ever-evolving. We have reviewed the current definition of intermediate-risk PE, including caveats, with preference towards the current ESC guidelines as a framework to evaluate patients and have discussed the role of cardiac biomarkers and imaging findings to support diagnosis. Previous metrics for RV dysfunction may not be as clear, reproducible, or predictive in defining intermediate-risk PE. Further studies examining echocardiographic and CT parameters are needed. Anticoagulation remains the cornerstone of therapy. While the role of catheter-directed therapies with thrombolysis and mechanical thrombectomy have recently gained attention, their specific role in individualized care and influence on patient outcomes requires further longitudinal study. Lastly, decision-making for patients with intermediate-risk PE can be nuanced, and the use of multidisciplinary PERTs is recommended to direct patient care.

## Figures and Tables

**Table 1:** Guidelines for classification of pulmonary embolism severity by society [4,9].

Guideline/Statement	Classification	Hemodynamic Status	Cardiac Biomarkers	RV Dysfunction on Imaging	Risk Score
American Heart Association (2011) [9]	Low	Stable	Negative	Negative	Not incorporated
	Intermediate	Stable	BNP > 90 pg/mL N-terminal pro-BNP > 500 pg/mL Troponin I > 0.4 ng/mL Troponin T > 0.1 ng/mL	RV dilatation (4-chamber RV/LV diameter > 0.9) on CT or ECHO RV systolic dysfunction on ECHO	
	High	Sustained hypotension (systolic BP of <90 mm Hg) for 15 min or requiring iatrogenic support. Pulselessness Persistent bradycardia (HR < 40)	Present	Present	
European Society of Cardiology (2019) [4]	Low	Stable	Negative	Negative	PESI I-II
	Intermediate-Low	Stable	Requires EITHER positive biomarkers OR RV dysfunction imaging. *(definitions below*)		Meets classification of PESI III-IV or sPESI ≥ 1 (see Table 2)
	Intermediate-High	Stable	N-terminal pro-BNP > 600 pg/mL Troponin I or T elevation, consider age-adjusted high-sensitivity cut-off values.	RV/LV diameter ratio ≥ 1.0 on CT or ECHO RV systolic dysfunction on ECHO (ex TAPSE < 16 mm)	
	High	Cardiac arrest Obstructive shock with end-organ hypoperfusion (systolic BP < 90 mmHg or vasopressors despite adequate filling status) Persistent hypotension (systolic BP < 90 mmHg or a systolic BP drop ≥ 40 mmHg for >15 min)	Present	Present	

Blood pressure (BP), brain natriuretic peptide (BNP), computed tomography (CT), echocardiogram (ECHO), heart rate (HR), Pulmonary Embolism Severity Index (PESI), tricuspid annular plane systolic excursion (TAPSE), right ventricle (RV).

**Table 2:** Pulmonary Embolism Severity Index scores: original and simplified [19,20].

Parameter	Original PESI Score	Simplified PESI Score (sPESI)
Demographics		
- Age	+ Age in years	1 point if >80 years old
- Male sex	+ 10 points	-
Medical Comorbidities		
- Cancer	+ 30 points	
- Congestive Heart Failure	+ 10 points	1 point
- Chronic Pulmonary Disease	+ 10 points	*1 point for chronic lung or heart disease*
Initial Clinical Assessment		
- Pulse: >110 bpm	+ 20 points	1 point
- Respiratory rate: >30 bpm	+ 20 points	-
- Systolic BP: <100 mmHg	+ 30 points	1 point
- Temperature: <36 °C	+ 20 points	-
- Altered mentation	+ 60 points	-
- Arterial oxygen saturation < 90%	+ 20 points	-
Interpretation of PESI vs. sPESI Risk Calculations		
Low-Risk Categories:	Class I (very low): <65 points	0 Points
Outpatient Management to be Considered	Class II: 66–85 points	
Moderate to Very High-Risk Categories: Inpatient Management Recommended	Class III (moderate): 86–105 points Class IV (high): 106–125 points Class V (very high): >125 points	≥1 point: estimated 30-day mortality risk 10.9%

**Table 3:** Summary of device-related trials for treatment of intermediate-risk pulmonary embolism.

Trial	Device	Study Design/Aims	Outcomes	Limitations
**Ultrasound-facilitated catheter-directed thrombolysis (US CDT)**				
ULTIMA [56] (2013)	EkoSonic MACH4e Endovascular System	Randomized controlled trial. N = 59 patients. Aims: USAT + AC versus AC alone in the reversal of RV dilatation in *intermediate-risk* PE patients.	USAT + AC was superior to AC alone in reversing RV dilatation at 24 h. No increase in bleeding complications between the two arms.	Small study population size. Limited follow-up to 24 h reviewing ECHO metrics alone; no comparison for clinical outcomes.
SEATTLE II [57] (2015)	EkoSonic Endovascular System	Prospective, single-arm, multicenter study using US CDT and low-dose fibrinolytic therapy. N = 150 patients with proximal PE (included massive and submassive PE). Aims: change in RV/LV ratio and PA systolic pressure from baseline at 48 + 6 h after procedure. Safety outcome of major bleeding within 72 h and recurrent PE, all-cause mortality, and procedural complications.	Mean RV/LV diameter decreased (1.55 at baseline to 1.13 at 48 h). Mean PASP decreased (51.4 mm Hg at baseline to 37.5 mm Hg) at the end of procedure. Safety: 17 major bleeding events within 30 days of the procedure observed in 15 patients (10%).	Lack of comparator group for full-dose systemic fibrinolysis, half-dose systemic fibrinolysis, or anticoagulation alone. Excluded patients with stroke/TIA within 12 months, patients with INR > 3.0, or serum creatinine > 2.0. Limited follow-up to 30 days post-procedure. Limited follow-up to 24 h reviewing ECHO metrics alone; no comparison for clinical outcomes.
OPTALYSE PE [62] (2018)	EkoSonic Endovascular System	Intermediate-risk PE patients, randomized to one of four groups of varying timeframes and concentrations of tPA infusion. N = 101. Aim: reduction in RV:LV ratio by CTA and embolic burden by modified Miller score on CTPA at 48 h.	Improvement in RV:LV ratio and modified Miller score was observed in all groups. Four patients experienced MBE, two being intracranial hemorrhage.	Small study population size. Unclear if improvement in CTPA metrics translate into short or long-term clinical benefits or adverse outcomes.
HI-PEITHO [63] (2022)	EKOS™ Endovascular System	Multi-center, prospective, randomized controlled trial for acute intermediate high-risk PE. Aim: EKOS + AC vs. AC alone for composite outcome of PE-related death, circulatory collapse, or non-fatal recurrence of PE.	Ongoing trial.	
Pharmacomechanical catheter-directed thrombolysis trials				
RESCUE [64] (2022)	Thrombolex-Bashir catheters	Multi-center, prospective trial. N = 109 patients. Aim: change in CTPA RV:LV ratio at 48 h and safety endpoint of serious adverse events in acute intermediate-risk PE.	RV/LV diameter ratio decreased by 0.56 (33.3%) and PA obstruction by refined modified Miller index was reduced (35.9%). Very low rate of adverse events or major bleeding (0.92%).	Small study population size. Lack of short-term or long-term clinical benefits or adverse outcomes data.
FLARE [65] (2019)	Inari FlowTriever catheters	Multi-center trial including symptomatic patients with RV/LV ratios > 0.9. N = 106 patients. Aim: Reduction in RV/LV ratio. Primary composite safety of device-related death, major bleeding, treatment related clinical decline, cardiac injury, or pulmonary vascular injury within 48 h.	RV/LV ratio was reduced by 0.38 at 48 h. Fourteen patients (13.2%) experienced serious adverse events at 30 days, with four (3.8%) occurring within 48 h of index procedure.	Small study population size. Lack of short-term or long-term clinical benefits or adverse outcomes data.
FLASH [66] (2022)	Inari FlowTriever catheters	Prospective, multi-center registry of high-risk or intermediate-risk PE. N = Goal of 250. Aim: composite endpoint for major adverse events including major bleeding, device-related bleeding, or death at 48 h.	Ongoing trial.	
PEERLESS [67] (2023)	Inari FlowTriever catheters	Prospective, multi-center, randomized controlled trial for intermediate or high-risk PE. N = goal of 550. Aims: composite endpoint for all-cause mortality, ICH, MBE, clinical deterioration, or ICU admission.	Ongoing trial.	
FLAME [68] (2023)	Inari FlowTriever catheters	Prospective, multi-center, non-randomized controlled trial for high-risk PE. N = 115 patients. Aims: composite of all-cause mortality, bailout to alternate thrombus retrieval strategy, MBE, or clinical decline.	Lower in-hospital adverse outcomes (17.0%) versus historical data (32.0%). Reduction in high-risk PE mortality compared to historical data.	Limited study population size. Unclear definitions of historical and context comparison groups.
EXTRACT PE [69] (2021)	Penumbra Indigo aspiration system	Prospective, single-arm, multi-center study with symptomatic acute PE ≤ 14 days, SBP ≥ 90 mm Hg, and RV/LV ratio > 0.9. N = 119 patients. Aims: safety and efficacy by RV/LV ratio reduction at 48 h for patients with submassive PE.	Mean RV/LV ratio reduction from baseline was 0.43 at 48 h post-procedure. Two (1.7%) of patients experienced a major adverse event. Rates were low for cardiac or pulmonary vascular injury, MBE, or device related death at 48 h.	Small study population size. Lack of short-term (beyond 48 h) or long-term clinical benefits or adverse outcomes data.

Anticoagulation (AC), blood pressure (BP), brain natriuretic peptide (BNP), computed tomography (CT), computed tomography pulmonary angiogram (CTPA), echocardiogram (ECHO), heart rate (HR), intracranial hemorrhage (ICH), intensive care unit (ICU), major bleeding event (MBE), pulmonary embolism (PE), Pulmonary Embolism Severity Index (PESI), systolic blood pressure (SBP), tricuspid annular plane systolic excursion (TAPSE), right ventricle (RV), ultrasound-assisted catheter-directed thrombolysis (USAT), ultrasound-facilitated catheter-directed thrombolysis (US CDT).
